# Different cerebrospinal fluid drainage methods and chronic hydrocephalus in patients with aneurysmal subarachnoid hemorrhage

**DOI:** 10.3389/fneur.2025.1564927

**Published:** 2025-04-14

**Authors:** Yang Zhou, Zhimin Liu, Huiqin Yan, Luyao Peng, Linshuang Chen, Wanyun Wu, Wei Luo, Yongkai Huang, Botao Wu

**Affiliations:** ^1^Department of Neurosurgery, Zhuzhou Hospital Affiliated to Xiangya School of Medicine, Central South University, Zhuzhou, China; ^2^Department of Anesthesiology, The Affiliated Zhuzhou Hospital of Xiangya Medical College, Central South University, Zhuzhou, China; ^3^Loudi Vocational and Technical College, Loudi, China

**Keywords:** aneurysmal subarachnoid hemorrhage, chronic hydrocephalus, cerebrospinal fluid drainage, risk factors, prognosis

## Abstract

**Background:**

Chronic hydrocephalus represents a common complication following aneurysmal subarachnoid hemorrhage (aSAH); however, the underlying mechanisms driving its pathogenesis remain incompletely understood. Furthermore, current evidence regarding optimal preventive strategies to mitigate hydrocephalus development remains controversial within the neurosurgical community.

**Objective:**

To investigate the efficacy of distinct cerebrospinal fluid (CSF) drainage modalities in mitigating the risk of developing chronic hydrocephalus among patients with aneurysmal subarachnoid hemorrhage (aSAH) through a comparative effectiveness study design.

**Method:**

The patients with aSAH treated in our hospital from January 2021 to January 2024 were analyzed retrospectively. Firstly, the related factors of chronic hydrocephalus in patients with subarachnoid hemorrhage were compared between patients with cerebrospinal fluid drainage and patients without cerebrospinal fluid drainage. Then, the related factors of hydrocephalus in patients with aneurysm subarachnoid hemorrhage with different cerebrospinal fluid drainage were compared. Univariate and multivariate logical regression analysis was used to determine the risk factors associated with chronic hydrocephalus.

**Result:**

Of the 246 hospitalized patients with aSAH, whether or not to receive cerebrospinal fluid drainage was associated with the formation of chronic hydrocephalus. A total of 67 patients (27.2%) developed hydrocephalus, of which 47 patients (34.8%) received cerebrospinal fluid drainage, while 20 (18%) patients developed chronic hydrocephalus. Of all IVH patients who received cerebrospinal fluid drainage, 34 (25.2%) received intermittent lumbar puncture drainage, 75 (55.5%) received continuous drainage in the lumbar cistern, and 26 (19.3%) received extraventricular drainage. Univariate analysis showed that different drainage methods had significant differences in postoperative chronic hydrocephalus in patients with aneurysmal subarachnoid hemorrhage (Purge 0.009). Multivariate Logistic regression analysis showed that different ways of cerebrospinal fluid drainage were independent risk factors for chronic hydrocephalus in patients with aneurysmal subarachnoid hemorrhage.

**Conclusion:**

Patients with aneurysmal subarachnoid hemorrhage must perform cerebrospinal fluid drainage. Among the three different drainage methods: lumbar puncture intermittent drainage, lumbar cistern continuous drainage, and extraventricular drainage, continuous lumbar cistern drainage is more effective in reducing the formation of chronic hydrocephalus.

## Introduction

Aneurysmal subarachnoid hemorrhage (aSAH) is a destructive disease, and the quality of life of aSAH survivors is often very low. The poor prognosis of this disease is mainly attributed to various complications, including cerebral infarction caused by cerebral vasospasm and secondary chronic hydrocephalus after aSAH (1). Severe initial symptoms, massive diffuse subarachnoid hemorrhage, and accompanying acute hydrocephalus during the onset of aSAH are associated with the occurrence of chronic hydrocephalus (2, 3).

The incidence of hydrocephalus in patients with aSAH is between 6 and 67 percent (4–8). According to the time after hemorrhage, the development of hydrocephalus after aSAH can be divided into three stages: acute (0–3 days), subacute (4–13 days), and chronic (14 days after aSAH) (9, 10). Although some patients may have self-limited acute hydrocephalus, other patients may have significant ventricular dilatation and increased intracerebral pressure, requiring cerebrospinal fluid drainage to relieve symptoms (11). The exact mechanism of acute hydrocephalus developing into chronic communicating hydrocephalus after subarachnoid hemorrhage is not completely clear. It is also important to note that acute hydrocephalus does not necessarily occur in all patients with chronic hydrocephalus. Studies have shown that the occurrence of hydrocephalus after aSAH involves a variety of mechanisms, including dynamic changes in cerebrospinal fluid, obstruction of arachnoid granules by blood products, and adhesion in the ventricular system (12). Many factors are associated with hydrocephalus after subarachnoid hemorrhage, including advanced age, hypertension, intraventricular hemorrhage, diffuse subarachnoid hemorrhage, aneurysms located in the posterior circulation, focal ischemic injury, ventricular enlargement on admission, poor Hunt, Hess and Fisher scores, symptomatic vasospasm, aneurysm rebleeding, and women (1, 13). In short, the development of hydrocephalus after aSAH involves a variety of mechanisms, and many factors are related to its occurrence. Further research is needed to fully understand the pathogenesis and risk factors of hydrocephalus in aSAH patients and to formulate effective prevention and management strategies.

It is preferred for patients with acute hydrocephalus or massive intraventricular hemorrhage. However, intermittent lumbar puncture drainage is also often used in patients with aSAH, mainly for patients with relatively mild conditions at admission and less hemorrhage in the ventricular system. At present, there is still no clear standard for the method of cerebrospinal fluid drainage after aSAH, and the best treatment is still controversial and further studied. In particular, there are limited studies on the differences between these three drainage methods in reducing the formation of hydrocephalus in aSAH patients. Therefore, the purpose of this study was to determine the risk factors for the formation of chronic hydrocephalus in aSAH and to compare the effects of EVD, LD, and intermittent lumbar puncture drainage in reducing the formation of chronic hydrocephalus.

## Methods

### Patient identification and selection

We reviewed all patients who received endovascular treatment in our hospital from January 2021 to January 2024. Inclusion criteria: 18–80 years old; diagnosis of aSAH; aneurysms caused by SAH on digital subtraction angiography (DSA), three-dimensional CT angiography, or magnetic resonance angiography in the medical center by computed tomography (CT) or lumbar puncture, which is the cause of subarachnoid hemorrhage; endovascular treatment was performed. Exclusion criteria: ruptured intracranial aneurysms caused by trauma and unexplained subarachnoid hemorrhage; microsurgical clipping or conservative treatment; loss of follow-up.

### Clinical parameters

The baseline data of the patients were recorded, including sex, age, smoking history, drinking history, hypertension history, diabetes history, coronary heart disease history, ruptured aneurysm history, Hunt Hess grade, GCS score on admission; imaging features of aneurysms, such as aneurysm size (maximum diameter) and location; and whether cerebrospinal fluid drainage and drainage methods were available after operation (lumbar puncture intermittent drainage, lumbar cistern continuous drainage and outdoor drainage). Postoperative complications, such as pulmonary infection, intracranial infection, and hydrocephalus. According to the imaging data at discharge and the head CT scan followed up for 3 months after discharge, patients with hydrocephalus and patients who needed temporary or permanent intraventricular drainage catheter were considered as chronic hydrocephalus. It is up to the attending neurosurgeon to decide whether or not to perform cerebrospinal fluid drainage and which drainage method to use. The interviewer was unaware of the situation.

This study has been approved by the Ethics Committee of the Affiliated Zhuzhou Hospital of Xiangya Medical College, Central South University.

### Outcome assessment

The main outcome was chronic hydrocephalus. Hydrocephalus refers to excessive cerebrospinal fluid in the ventricle. The diagnosis of hydrocephalus is mainly based on clinical manifestations and neuroimaging examination. Hydrocephalus may be characterized by headache, nausea, vomiting, coma, and/or gradual slowing of cognitive and motor activity, gait ataxia, cognitive impairment, and urinary incontinence (14). The diagnostic neuroimaging examination of hydrocephalus is calculated based on a CT scan, the width of the third ventricle, and the value index of internal media (CMI=B/A, where A is the width of the outer skull and B is the width of the lateral ventricle). The CMI value higher than 0.25 and the width of the third ventricle more than 7 mm were regarded as pathological (15). The diagnosis of hydrocephalus was confirmed by radiology and diagnosed by two experienced neurosurgeons.

### Data analysis

**Figure 1:**
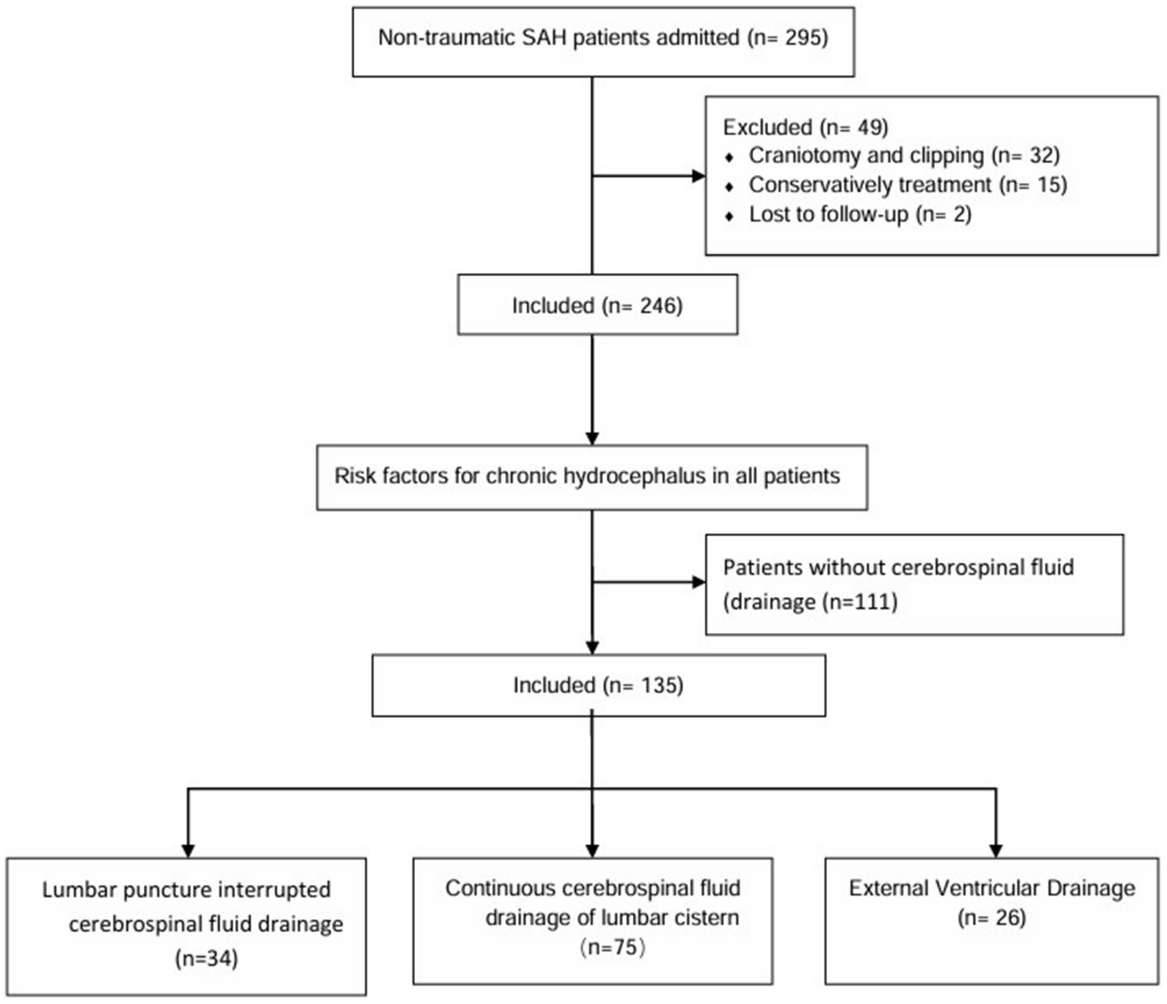
Flow diagram.

SPSS 26.0 software (IBM, Almonk, NY) was used for statistical analysis. The measurement data by normal distribution are represented by ±s, and those that do not accord with normal distribution are represented by median and quartile [M (P25, P75)]. T-tests or rank sum tests are used for comparison between groups. The count data were expressed as the number and percentage of cases [*n* (%)] and were compared between groups using tests or Fisher exact tests. Firstly, the risk factors related to the formation of chronic hydrocephalus in all patients were analyzed and compared. Then the differences among patients with aneurysmal subarachnoid hemorrhage treated with different methods of cerebrospinal fluid drainage were analyzed and compared, and the influencing factors of chronic hydrocephalus in patients with different drainage methods were analyzed by multivariate logistic regression. *p* < 0.05 is defined as statistically significant (see Figure 1).

## Results

### Baseline characteristics of the study cohort for all patients

A total of 246 hospitalized patients with aSAH were included in this study. As shown in Table 1, the incidence of chronic hydrocephalus is 27.2% (*n* = 67). GCS Score (*p* < 0.001), Hunt Hess grading (*p* < 0.001), whether cerebrospinal fluid drainage was performed after surgery (*p* = 0.003) There were statistically significant differences in pulmonary infection (*p* < 0.0010), intracranial infection (*p* = 0.001), length of hospital stay (*p* = 0.001), and mRS score at 3 months (*p* < 0.001).

**Table 1:** Compare the demographic, clinical, aneurysm, and treatment characteristics of all patients.

	Hydrocephalus		
Characteristic	Yes (*n* = 67)	No (*n* = 179)	*p* value
Age, years, mean (SD)	62.6 (9.8)	59.5 (9.6)	**0.027**
Female, *n* (%)	34 (50.7)	87 (48.6)	0.765
Smoking history, *n* (%)	7 (13.4)	26 (14.5)	0.404
History of drinking alcohol, *n* (%)	8 (11.9)	19 (10.6)	0.767
Hypertension, *n* (%)	49 (73.1)	101 (56.4)	**0.017**
Heart disease, *n* (%)	5 (7.5)	18 (10.1)	0.534
Diabetes, *n* (%)	9 (13.4)	17 (9.5)	0.371
History of aSAH, *n* (%)	6 (9.0)	10 (5.6)	0.340
GCS score, mean (SD)	14 (2.7)	11.2 (3.7)	**<0.001**
Hunt-Hess grade, mean (SD)			**<0.001**
1–3	47 (70.1)	166 (92.7)	
4–5	20 (29.9)	13 (7.3)	
Aneurysm location			0.414
Anterior circulation, *n* (%)	54 (80.6)	152 (84.9)	
Posterior circulation, *n* (%)	13 (19.4)	27 (15.1)	
Aneurysm size (mm), mean (SD)	5.5(2.5)	5.1 (1.7)	0.245
Aneurysm neck width	4.2(1.9)	3.8(1.1)	**0.029**
External cerebrospinal fluid (CSF)			**0.003**
No	20 (29.9)	91 (50.8)	
Yes	47 (70.1)	88 (49.2)	
Pulmonary infection, *n* (%)	33 (49.3)	41 (22.9)	**<0.001**
Intracranial infection, *n* (%)	14 (20.9)	12 (6.7)	**0.001**
DCI, *n* (%)	3 (4.5)	4 (2.2)	0.346
Length of stay, mean (SD)	19.7 (14.6)	13 (6.0)	0.001
mRS score at follow-up, *n* (%)			**<0.001**
0–3	165 (92.2)	41 (61.2)	
4–6	26 (38.8)	14 (7.8)	

Remark: The value in bold indicates *p* < 0.05. aSAH, aneurysmal subarachnoid hemorrhage; SD, standard deviation; CSF, External cerebrospinal fluid. Divide patients into two groups based on the presence or absence of chronic hydrocephalus. Groups were compared using the χ^2^ test, Fisher exact test, or student t test.

### Baseline characteristics of a study cohort of patients who received cerebrospinal fluid drainage

A total of 135 hospitalized patients with aSAH received cerebrospinal fluid drainage. As shown in Table 2, among all IVH patients who received cerebrospinal fluid drainage, 34 patients (25.2%) received lumbar puncture intermittent drainage, 75 patients (55.5%) received continuous drainage in the lumbar cistern, and 26 patients (19.3%) received intraventricular drainage. The two groups were evaluated based on age (*p* = 0.048), history of hypertension (*p* = 0.011), GCS score at admission (*p* < 0.001), HuntHess grading (*p* = 0.009), postoperative cerebrospinal fluid drainage (*p* = 0.009), and intracranial infection (*p* = 0.023) There were statistically significant differences in hospitalization duration (*p* < 0.001) and mRS score at 3 months (*p* < 0.001).

**Table 2:** Comparison of demographic, clinical, aneurysm, and treatment characteristics of patients with three different types of cerebrospinal fluid drainage.

	Hydrocephalus		
Characteristic	Yes (*n* = 47)	No (*n* = 88)	*p* value
Age, years, mean (SD)	63.5 (8.1)	60.0 (10.3)	**0.048**
Female, *n* (%)	21 (44.7)	51 (58.0)	0.141
Smoking history, *n* (%)	6 (12.8)	11 (12.5)	0.965
History of drinking alcohol, *n* (%)	6 (12.8)	9 (10.2)	0.655
Hypertension, *n* (%)	37 (78.7)	50 (56.8)	**0.011**
Heart disease, *n* (%)	3 (6.4)	7 (8.0)	0.740
Diabetes, *n* (%)	7 (14.9)	9 (10.2)	0.424
History of aSAH, *n* (%)	4 (8.5)	2 (2.3)	0.094
GCS score, mean (SD)	10.8 (3.7)	13.1 (3.6)	**<0.001**
Hunt-Hess grade, *n* (%)			**0.009**
1–3	13 (44.8)	75 (70.8)	
4–5	16 (55.2)	31 (29.2)	
Aneurysm location			0.513
Anterior circulation, *n* (%)	41 (87.2)	73 (83.0)	
Posterior circulation, *n* (%)	6 (12.8)	15 (17.0)	
Aneurysm size (mm), mean (SD)	5.5 (2.7)	5.2 (1.7)	0.450
Aneurysm neck width	4.1 (1.5)	3.7 (1.1)	0.392
External cerebrospinal fluid (CSF)			**0.009**
Lumbar puncture	18 (38.3)	16 (18.2)	
LD	18 (38.3)	57 (64.8)	
EVD	11 (23.4)	15 (17.0)	
Pulmonary infection, *n* (%)	28 (31.8)	22 (46.8)	0.086
Intracranial infection, *n* (%)	14 (29.8)	12 (13.6)	**0.023**
DCI, *n* (%)	2 (4.3)	3 (3.4)	0.804
Length of stay, mean (SD)	21.7 (16.4)	15 (6.0)	**<0.001**
mRS score at follow-up, *n* (%)			**<0.001**
0–3	26 (55.3)	74 (84.1)	
4–6	21 (44.7)	14 (15.9)	

Remark: The value in bold indicates *p* < 0.05. aSAH, aneurysmal subarachnoid hemorrhage, IVH, intraventricular hemorrhage; SD, standard deviation; EVD, external ventricular drainage; LD, lumbar drainage. CSF, External cerebrospinal fluid. Divide patients into two groups based on the presence or absence of chronic hydrocephalus. Groups were compared using the χ^2^ test, Fisher exact test, or student t-test.

### Factors affecting the formation of hydrocephalus in patients

The logistic regression equation was used to analyze the influencing factors of chronic hydrocephalus formation in patients with aneurysmal subarachnoid hemorrhage. Factors with *p* values less than 0.05 included age, history of hypertension, GCS score at admission, HuntHess grading, different cerebrospinal fluid drainage methods, intracranial infection, length of hospital stay, and mRS score at 3 months. Different cerebrospinal fluid drainage methods are independent risk factors for the formation of chronic hydrocephalus (see Table 3).

**Table 3:** Multi-factor model of factors affecting the formation of hydrocephalus in patients with different cerebrospinal fluid drainage methods.

Variable	Odds ratio	95%CI	*p* value
Age	1.025	0.976–1.076	0.329
GCS	0.961	0.685–1.350	0.821
HuntHess grade	1.386	0.508–3.783	0.524
External cerebrospinal fluid (CSF)	0.440	0.205–0.945	**0.035**
Hydrocephalus	2.490	0.933–6.641	0.068
Length of hospital stay	1.053	0.999–1.109	0.055
Intracranial infection, *n* (%)	1.816	0.540–6.105	0.335
mRS score at follow-up, *n* (%)	0.898	0.170–4.731	0.899

Remark: The values in bold are statistically significant (*p* < 0.05). OR, Odds Ratio; CI, confidence interval.

Compare the effects of three drainage methods, namely lumbar puncture intermittent drainage, lumbar cistern drainage, and intraventricular drainage, on the formation of chronic hydrocephalus in patients with aneurysmal subarachnoid hemorrhage. There was a statistically significant difference (*p* = 0.035) in the formation of chronic hydrocephalus among patients who received different drainage methods. Although age, history of hypertension, GCS score at admission, Hunt Hess grading, different cerebrospinal fluid drainage methods, intracranial infection, length of hospital stay, and mRS score at 3 months were all associated with the formation of hydrocephalus in univariate analysis, when we included these possible influencing factors in the multivariate logistic regression model, we found significant differences between the two groups of patients receiving different cerebrospinal fluid drainage methods and the formation of chronic hydrocephalus. Cerebrospinal fluid drainage methods are independent risk factors for the formation of chronic hydrocephalus.

## Discussion

Pathophysiological mechanisms of chronic hydrocephalus following aneurysmal subarachnoid hemorrhage (aSAH) are postulated to involve fibrotic adhesions in the subarachnoid space and obstruction by hemoglobin degradation products, which impair cerebrospinal fluid (CSF) dynamics through both mechanical blockage and ependymal damage (1). Emerging evidence from prospective cohort studies demonstrates that hemorrhage burden quantification metrics—including basal cistern clot volume (Fisher grade), intraventricular hemorrhage score, and parenchymal hematoma diameter—exhibit significant associations with ventriculoperitoneal shunt dependence rates (adjusted OR 2.1–4.3, *p* < 0.01) (16–18). Notably, acute hydrocephalus requiring emergent external ventricular drainage independently predicts chronic progression with 78% sensitivity in recent multicenter analyses (17). However, systematic reviews highlight persistent knowledge gaps regarding modifiable risk factors, as current predictive models account for only 61% of outcome variance in validation cohorts (19–21). To address these critical gaps, this study aims to: (1) elucidate dose–response relationships between cisternal blood clearance kinetics and chronic hydrocephalus development, and (2) identify evidence-based strategies for risk stratification through multimodal monitoring of CSF inflammatory biomarkers.

Previous studies have demonstrated the efficacy of cerebrospinal fluid (CSF) drainage in mitigating chronic hydrocephalus formation following aneurysmal subarachnoid hemorrhage (aSAH) (22–24). Nevertheless, academic consensus remains divided regarding the mechanistic superiority of continuous versus intermittent drainage protocols. A Japanese cohort study posits that intermittent CSF drainage preserves arachnoid membrane adhesion within the subarachnoid space, thereby reducing chronic hydrocephalus incidence (25). Contrarily, aggregated data from two German tertiary centers revealed contradictory evidence in their observational cohort analysis (26). Our retrospective analysis revealed a paradoxical association: 34.8% (47/135) of patients receiving CSF drainage developed chronic hydrocephalus, compared to 18% (20/111) in the non-drainage cohort (Table 1). Notably, admission Glasgow Coma Scale (GCS) scores exhibited significant divergence between groups (chronic hydrocephalus: median 8 vs. non-hydrocephalus: median 13, *p* < 0.01). Hunt-Hess stratification further demonstrated differential outcomes: 22.1% (47/212) of grades 1–3 patients developed chronic hydrocephalus versus 60.6% (20/33) in grades 4–5. This severity-dependent pattern aligns with existing literature documenting acute hydrocephalus prevalence (100%) in critical-grade patients necessitating intraventricular drainage preoperatively. The elevated infection rates observed in drainage recipients (intracranial: 12.6% vs. 3.6%; pulmonary: 18.5% vs. 5.4%) may reflect prolonged external drainage duration in severe cases. While seemingly contradictory to previous findings (7, 27, 28), this discrepancy likely stems from inherent selection bias in retrospective studies, where drainage allocation inherently correlates with baseline disease severity. Subgroup analysis of drainage modalities (continuous vs. intermittent) revealed no significant outcome differences (*p* = 0.32), suggesting protocol selection exerts less impact than initial hemorrhage severity in chronic hydrocephalus pathogenesis.

Our study compared the efficacy of intermittent lumbar puncture drainage (ILPD), continuous lumbar drainage (CLD), and external ventricular drainage (EVD) in reducing the incidence of chronic hydrocephalus (CH) following aneurysmal subarachnoid hemorrhage (aSAH). As summarized in Table 2, CH occurred in 18 patients (52.9%) undergoing ILPD, compared to 18 patients (24%) treated with CLD. This discrepancy contrasts with findings by Yamanaka et al. (25), who reported superior outcomes with CLD. Notably, our data suggest that CLD may mitigate CH risk by continuously removing hemorrhagic cerebrospinal fluid (CSF), thereby reducing obstruction of arachnoid granulations by blood-derived macromolecules. However, the pathophysiological basis for ventricular dilation without concomitant subarachnoid space expansion in hydrocephalus remains unresolved. While adults produce 300–400 mL of CSF daily, our protocol limited drainage to ≤150 mL/day, minimizing risks of subarachnoid space desiccation. Clinical decision-making between CLD and ILPD depended on subarachnoid hemorrhage volume: CLD was preferred for high-volume bleeding, whereas ILPD was reserved for low-volume cases. Among EVD-treated patients, 11 (42.3%) developed CH. Although EVD also employs continuous CSF drainage, its inferior efficacy relative to CLD may reflect confounding by baseline severity. Patients requiring EVD exhibited higher Hunt-Hess grades and lower Glasgow Coma Scale (GCS) scores at admission, indicating more severe neurological compromise. Furthermore, preexisting acute hydrocephalus necessitating EVD likely predisposed these patients to CH, consistent with Adams et al.’s (7) identification of EVD and acute hydrocephalus as independent CH risk factors. Multivariable logistic regression adjusted for age, hypertension history, Hunt-Hess grade, GCS score, intracranial infection, hospitalization duration, and 3-month modified Rankin Scale (mRS) confirmed CSF drainage modality as an independent predictor of CH. Current reliance on admission imaging and clinical parameters for drainage selection may overlook dynamic pathophysiological changes. Future studies should prioritize standardized protocols integrating real-time biomarkers or advanced imaging to optimize drainage strategy selection.

Based on the aforementioned analytical findings, it is recommended that patients with aneurysmal subarachnoid hemorrhage (aSAH) receive aggressive cerebrospinal fluid (CSF) drainage postoperatively, with continuous lumbar drainage representing a reliable therapeutic approach. Although CSF drainage carries a potential risk of intracranial infection, this risk can be mitigated through standardized protocols, and the clinical benefits of lumbar drainage outweigh these concerns in patients with substantial hemorrhage volumes. For critically ill patients presenting with Hunt-Hess grade IV-V severity, intraventricular drainage should be prioritized. This intervention achieves multiple therapeutic objectives: (1) mitigating chronic hydrocephalus risk through efficient CSF turnover; (2) rapidly lowering intracranial pressure via blood-tinged CSF evacuation; (3) alleviating acute hydrocephalus manifestations; and (4) reducing vascular spasm incidence by diminishing hemoglobin degradation product exposure (29). The selection of drainage modality should be determined through comprehensive evaluation of admission clinical status, incorporating both intracranial hypertension manifestations and neuroimaging characteristics of acute hydrocephalus. Notably, while a subset of patients without CSF drainage intervention avoided chronic hydrocephalus development, proactive CSF drainage remains warranted in aSAH management. Strategic optimization of drainage duration (typically 5–7 days) is advised to balance therapeutic efficacy with infection prevention.

### Limitations

Potential limitations to our retrospective review include those that are inherent to all retrospective analyses. Additionally, whether the cerebrospinal fluid drainage and which drainage method is adopted is determined by the attending neurosurgeon, with no standard regimen or dosage.

## Conclusion

Continuous lumbar cistern drainage should be actively performed in patients with aneurysmal subarachnoid space after embolization, and outdoor drainage should be actively performed in severe patients or patients with acute hydrocephalus.

## Data Availability

The raw data supporting the conclusions of this article will be made available by the authors, without undue reservation.
